# From cruel optimism to foreclosed futures: The emotional and temporal dimensions of enduring precarity among young adults in Barcelona

**DOI:** 10.1177/02637758251364077

**Published:** 2025-08-06

**Authors:** Santiago del Río

**Affiliations:** 1 University of Manchester, UK

**Keywords:** Youth precarity, urban precarity, emotional experiences, temporality, foreclosed futures, cruel optimism

## Abstract

This paper contributes to geographical research on precarity by exploring how emotional responses to enduring insecurity shape young people's temporal horizons, spatial belonging, and sense of possibility. Drawing on qualitative research in Barcelona, I advance multi-dimensional perspectives on precarity by showing how precarious entanglements – where the material, affective, temporal, and spatial become embodied – shape the contours of potentiality and futurity. I conceptualise ‘foreclosed futures’ as a temporal–affective structure that emerges from, yet unsettles, the logic of Lauren Berlant's ‘cruel optimism’. For a generation shaped by austerity, the projection of present structural barriers to upward mobility into the future precludes the imagination of prosperous futures, fostering hopelessness, disillusionment, and nostalgia for lost stability. To unpack this dynamic, I illuminate how persistent precarity resignifies the urban – not as a site of potentiality, but as one of constraint leading to foreclosure of potentiality. I then demonstrate how a sense of inevitable downward mobility and social decline alters the emotions and temporalities tied to post-war ideals of security woven into cruel optimism. Finally, I show how apprehension towards insecure futures anchors young people in present-oriented survival, fostering pragmatic and pessimistic attitudes, concluding with an analysis of the socio-political implications of foreclosed futures.

## Introduction

Precarity emerges as a persisting aspect of the daily realities of young and not-so-young people, shaping their sense of themselves, their emotional landscapes, journeys into adulthood, and their imaginaries of the future. Emerging research (Furlong et al., 2017; Lorey, 2015) illuminates how in the Global North precarity is becoming the norm, rather than an anomaly, with austerity increasingly being subjectively internalised by young people as an unending reality (del Río et al., 2025). Nascent research exploring the emotional geographies of precarity and austerity (Hall, 2023; Pimlott-Wilson, 2017; Raynor, 2021; van Lanen, 2021) elucidates how the normalisation of insecurity is emotionally experienced. Expanding upon this work, this paper advances research on geographies of precarity (Ferreri, 2021; Strauss, 2017) with a particular emphasis on how the emotions stemming from perceived unending insecurity shape young people's temporal landscapes and sense of possibility. The conceptual approach and findings of this paper also deepen current multi-dimensional understandings of precarity (Alberti et al., 2018; Ferreri, 2021; Lorey, 2015; Millar, 2007; Strauss, 2017) by delving into precarious entanglements where material, affective, temporal, and spatial dimensions intersect and become embodied, shaping the contours of potentiality and futurity.

This paper is part of the Austerity and Altered Life-Courses project and draws on 39 oral history in-depth interviews supported by creative methods specifically developed to facilitate conversations about the future with young people aged 19–35 in the Metropolitan Area of Barcelona.^1^ The Spanish context is notably significant, as an entire generation of young Spaniards has only known precarious labour markets and austere material conditions – a phenomenon that curtails their autonomy in making life decisions, such as leaving the parental home or having children. In exploring how variegated, path-dependent politico-economic trajectories shape young people's emotions, ambitions and dreams, I demonstrate how, in Spain, extreme youth precarity, combined with objective downward mobility, shapes young adults’ attitudes towards the future.

In the first part of the paper, I expand upon del Río et al.'s (2025) notion of ‘foreclosed futures’, a term that indicates how enduring austerity, understood as an embodied and lived policy regime (Hall, 2022), increasingly produces long-term experiences of precarity beyond youth, reshaping normative ideas of life-course transitions. I conceptualise foreclosed futures as a temporal–affective formation that emerges from the sense of precarity as a permanent condition, generating apprehension towards future-oriented imagination, narrowing horizons of possibility, and reframing security through the lens of nostalgia and loss. I frame ‘foreclosed futures’ as a development that both emerges from and unsettles the temporal–affective structure of Berlant's ‘cruel optimism’. In 2006, before the onset of the 2008 global financial crisis and the ensuing long decade marked by austerity, Berlant (2006) first coined the concept of ‘cruel optimism’ to illuminate the ‘fraying relation between post-Second World War state/economic practices and certain postwar fantasies of the good life endemic to liberal, social democratic, or relatively wealthy regions’ (2011: 15). Cruel optimism denotes the damaging pursuit of unachievable aspirations disavowed by neoliberalism and austerity. Acknowledging ‘cruel optimism’ as an invaluable conceptual tool and recognising its ongoing relevance, this paper explores whether it fully aligns with how young people in Barcelona emotionally navigate life and envision the future.

Exploring this, the second part of paper interrogates how a generation raised during austerity interacts with both Western post-war ideals of ‘the good life’ and neoliberal notions of flexibility, autonomy, and success engendering precarity. This includes investigating how young people make sense of their inability to meet aspirations bound to cruel optimism and how the normalisation and anticipation of precarity affects their emotional experiences, sense of time, and capacity to imagine other possible futures. Drawing on the participant's narratives, I investigate how multi-dimensional experiences of persistent precarity redefine spatial belonging, transforming the urban from a realm of potentiality into one of impotence and foreclosed possibility. I then examine how enduring precarity cultivates a sense of inevitable social decline, where security is not envisioned through the promise of hopeful futures, but as part of an imagined nostalgic past. Finally, I explore how apprehension towards uncertain futures anchors participants in present-oriented survival, fostering pragmatic and sceptical attitudes, and conclude with a brief reflection on the socio-political implications of foreclosed futures.

## A multi-dimensional understanding of enduring precarity

Precarity, precariousness, and precarisation are key approaches for examining precarious life, shaping evolving debates across the social sciences. A comprehensive review of these cognate concepts – which often overlap and are not always used consistently – falls beyond the scope of this paper (see Lorey, 2015). Instead, I seek to advance approaches that bridge the ideas of precarity as the emergence of insecure labour in post-Fordist economies (Bourdieu, 1998; Castel, 2002; Kalleberg, 2009), precariousness as an ontological condition and lived experience (Butler, 2004), and precarisation as a mode of governance (Lorey, 2015). Of the three terms, precarity is the most widely used. Originating in French (précarité), Spanish (precariedad), and Italian (precarietà), it initially described the structural shift from Fordist labour regimes to insecure, casual, and low-wage work – driven by neoliberal restructuring, deregulation, and the erosion of social protections. This concept emerged to analyse the rise of atypical employment and the destabilisation of the Fordist wage–labour mode (Castel, 2002; Kalleberg, 2009). The second concept – precariousness – expands beyond labour to describe an increasingly widespread existential condition defined by uncertainty, insecurity, and vulnerability. Butler (2004) has advanced the idea of precariousness as a shared human experience grounded in inter-dependency and vulnerability, which is unevenly distributed through socio-political exclusion, rendering certain social groups more disposable. The third term – precarisation – is conceptualised by Lorey (2015) as a form of governmentality based on the normalisation and acceptance of precarity as a self-chosen paths geared by the seductive power of discourses autonomy and self-realisation. The internalisation of these discourse results in individuals governing themselves in ways that align with neoliberal logics, effectively becoming both subjects and agents of their own precarisation.

Bringing these perspectives together, sociological and geographical research conceptualises precarity as a multi-dimensional and lived process, shaped by intersecting socio-political forces that extend beyond the labour market and emerge across multiple domains of life (Alberti et al., 2018; Ferreri, 2021; Millar, 2007; Strauss, 2017). The approach I adopt contributes to this body of work by bridging the structural, lived, and governmental dimensions of precarity. I illuminate how multi-scale dynamics produce place-based assemblages of material insecurity that are felt in and through space–time, shaping emotions, aspirations, dreams, and spatial belonging. In this vein, Ferreri (2021: 165) argues that the intersection of work, place, and life insecurity embeds precarious life into spatial processes that deeply transform the urban. Drawing on Lorey (2015), Ferreri links processes of self-precarisation to the seductive power of temporary urbanism: ‘an urban model that glorifies ephemerality and disruption over continuity and permanence’ (2021: 146). She argues that this form of urbanism generates an ambivalence marked by fascination for ephemeral urban practices and resignation to precarious urban conditions. In linking the normalisation and anticipation of precarity to the foreclosure of alternative futures, Ferreri notes (2021: 165),

When urban inhabitants anticipate precarity, they participate in the perpetuation of a condition of impotence in relation to the dynamics that produce and reproduce cities and urban spaces. Impotence is here not understood as a lack of power but rather as the absence of potentiality.

As I show in detail in the empirical sections, the idea of impotence not only understood as frustration and powerlessness but as the foreclosure of horizons of possibility is particularly important to understand the temporal and emotional dimensions of enduring precarity. At the same time, I examine whether the idea self-precarisation, shaped by the internalisation and alluring power of discourses on flexibility and autonomy, is either reinforced or disrupted when precarity becomes an unending temporal horizon.

Temporality is a key aspect in conceptualisations of precarity. Ettlinger (2007: 320) defines precarity as a ‘condition of vulnerability relative to contingency and the inability to predict’. Similarly, Rossiter and Neilson (2005: para. 5) conceptualise it as ‘an interminable lack of certainty, the condition of being unable to predict one's fate or having some degree of stability on which to construct a life’. While precarity is often conceptualised as the erosion one's ability to predict and plan, there is scope to advance a more nuanced temporal understanding of precarity – especially in relation to how the emotions stemming from ontological precariousness shape imaginaries of the future and temporal understanding of material security.

Research recognises that labour precarity has become the objective new normal in increasingly elongated young adulthoods. Furlong et al. (2017) argue that precarity is only truly ‘normalised’ when it is subjectively internalised – a pattern increasingly evident in studies on young people (Berry and McDaniel, 2020). Going further, emerging literature positions the normalisation of precarity as a persistent and potentially permanent condition. Specifically, I draw on Hall's (2022) conceptualisation of austerity and del Río et al.’s (2025) notion of foreclosed futures to deepen current temporal understandings of precarity. Hall views austerity not as a policy regime with a defined beginning and end, but as a multi-faceted, lived process with its own social and embodied life, one that can endure indefinitely and may be experienced as unending. Informed by this, del Río et al. introduce the notion of ‘foreclosed futures’ to illustrate how enduring austerity may not only be experienced as an elongation of precarity linked to delayed transitions to adulthood – an idea that pre-supposes the eventual achievement of stability, albeit later in life. Instead, foreclosed futures refer to a permanent denial of material and ontological security seizing young people's autonomy to make and imagine alternative futures. In doing so, del Río et al. recognise the possibility that precarity increasingly manifest and is internalised as a permanent state, re-examining life-course theories that theorise youth precarity as ‘arrested’ or ‘emerging adulthood’ (Arnett, 2014; Cote, 2000). In empirically exploring del Río et al.'s conceptual approach, I explore ‘foreclosed futures’ as temporal–affective structure shaped by the sense that material security belongs to a past that is irrevocably lost.

The idea of precarity as loss aligns with Allison's (2013: 7) insights. Highlighting the Western-centric bias behind this idea, she argues that precarity is best understood as the loss of a type of security that was never universal but fleeting and unevenly distributed: ‘the loss of something that only certain countries, at certain historical periods, and certain workers ever had in the first place’. Similarly, Neilson and Rossiter (2008: 54) highlight that precarity has long been the norm in the history of capitalism: ‘if we look at capitalism in a wider historical and geographical scope, it is precarity that is the norm and not Fordist economic organization’. Fordism marked an unprecedented rise in living standards working-class Western households, combining universal public services with exclusionary forms of redistribution. Security was largely accessed through the family wage system, which privileged white, male breadwinners. Unmarried women, the LGBT community, racialised people, and migrants were often denied secure jobs, social security, mortgages, and family benefits (Butler, 1997; Fraser, 2013). In many Western countries, married women were legally classified as dependent only accessing social security entitlements through their husbands’ employment status (Abramovitz, 2017; Fraser, 2013), while many white male workers remained non-unionised and insecure. Romanticised images of the post-war period as a time of egalitarian security elide the exclusionary and uneven elements inherent in Fordism, not to mention the ongoing (neo)colonial dispossession that supports Western welfare. And yet, as Berlant illustrates through the concept of cruel optimism (2011), powerful ideals of the ‘good life’ remain tethered to this brief moment in capitalist history, continuing to shape our desires and understandings of material security, stability, and life progression.

## From cruel optimism to foreclosed futures

Berlant's concept of cruel optimism captures how affective attachments to post-war Fordist ideals are not experienced as loss, but rather as a damaging hope for a better future – one imagined through post-war ideals of the good life, now disavowed by neoliberalism. Her writings on ‘cruel optimism’ (2006, 2007, 2011) capture a complex affect-driven temporality where everyday lives in precarious contemporary contexts are animated by the conviction of a better future imagined through anachronistic post-war ideals. In other words, present-day precarity becomes bearable through emotional attachment to a secure past, projected forward into an imagined – yet foreclosed – better future. My conceptualisation of enduring precarity as a force shaping both emotional and temporal attachments stems from, yet also moves beyond, Berlant's notion of cruel optimism. Eighteen years after Berlant's brilliant concept was coined, with austerity and precarity becoming internalised as the new normal, it is pertinent to ask whether young people's lives are driven by cruel desires that are obstacles to their flourishing or whether, by contrast, they have abandoned hope for the achievement of security. Against this backdrop, I explore how a generation raised in the era of austerity relates to post-war ideals of ‘the good life’. How do young people understand, articulate, and navigate their inability to achieve aspirations tied to cruel optimism? How is the increasing normalisation of precarity as a permanent condition felt, and how does it shape young people's ability to imagine other futures?

In addressing these questions, I develop del Río et al.'s notion ‘foreclosed futures’ as a contemporary development of Berlant's idea of ‘cruel optimism’ – specifically, as a temporal–affective dimension of precarity that transforms the emotions and temporalities animating cruel optimism. Grounded in the narratives of this research, I explore how extreme precarity and lack of continuity have eroded the appeal of neoliberal ideals of flexibility and autonomy, fuelling nostalgic attachments to post-war security. In this sense, notions of the good life tied to cruel optimism continue to inform dominant understandings of security. However, the perception of precarity as a permanent condition reconfigures the emotions of temporalities linked to these longings. Unlike in cruel optimism, longings for security are not envisioned as hopeful futures but as ‘cruel nostalgia’ for a past perceived as irrevocably lost.

Emerging research in human geography has begun to capture the emotional rupture produced by unfulfilled promises of a better future, shedding light on the affective dimensions of precarity (Marcu, 2019; Raynor, 2021; van Lanen, 2021; Wilkinson and Ortega-Alcázar, 2019). This research shows that under conditions of persistent austerity people feel less and less in control of their lives. In studying the experiences of mobility of qualified young eastern Europeans in Spain, Marcu (2019: 926) shows that prolonged precarity in Spain turns the aspirations linked to upward mobility into ‘destabilization, despair and loss of direction’. In the UK, Pimlott-Wilson (2017) delves into the disconnection between neoliberal policy and the everyday realities of working-class young people highlighting that aspirations to professional success have a great emotional impact on young people who find these goals unattainable. In exploring the relationship with hope of ‘women on benefits’ in Northeast England, Raynor (2021) argues that in the face of normalised decline, hopes are not invested in upward mobility but in pragmatically maintaining a normalised precarious position. Similarly, Van Lanen (2021) explores the emotional impact of enduring austerity among Irish youth, noting that some of his participants abandoned long-term planning, accepted unending austerity, or retreated to focusing solely on the day-to-day.

My conceptualisation of foreclosed futures extends research in emotional geographies through a closer examination of the temporal formations that arise from felt experiences of precarity. In particular, I use this concept as a conduit to explore the impact of prolonged precarity on future-oriented imagination and spatial belonging, while beginning to illuminate its socio-political implications. Emphasising the situated nature of these dynamics, I propose ‘foreclosed futures’ as a key biographical feature of Spanish young adults – while acknowledging that such experiences may differ in contexts shaped by distinct, path-dependent political economies. In this sense, further empirical research to evaluate the concept's broader relevance is needed. For example, scholars studying the emotional geographies of precarity in the Global South (Alacovska et al., 2021; Pettit, 2019) argue that, for the majority of the world, precarity is not a contemporary phenomenon reshaping deep-seated emotional and temporal textures but a historically persistent condition of life. In contrast to objective downward mobility and the increasing sense of social decline in the Global North, these scholars (Alacovska et al., 2021; Pettit, 2019) highlight how optimistic discourses about ‘emerging economies’, ‘development’, ‘growth’, and ‘economic rising’ in the Global South ambivalently generate both cruel and generative hope among young people. For example, in his study of educated underemployed men in Egypt, Pettit (2019) highlights the prominence of cruel optimism in reinforcing the logic of neoliberal discourses on self-responsibility, individual failure and self-blame. By contrast, as I examine below, lived experiences of foreclosed futures do not manifest as neoliberal self-blame or self-precarisation, but instead reflect a critical awareness of the structural barriers limiting young people's autonomy while reshaping the meaning of the urban as a site of potentiality and upward mobility.

The anticipation and normalisation of precarity has led the participants in this research to relinquish the future-oriented attachments typically associated with cruel optimism. As Berlant (2007: 33) herself notes referring to the potentially devastating consequences of this, ‘if the cruelty of an attachment is experienced by someone/some group, even in disavowed fashion, the fear is that the loss of the object/scene of promising itself will defeat the capacity to have any hope about anything’. Somewhere else, she asks herself (Berlant, 2011: 2) ‘What happens when those fantasies start to fray – depression, dissociation, pragmatism, cynicism, optimism, activism, or an incoherent mash?’ In engaging with Berlant's questions, in what follows, I illuminate how broken promises and unmet expectations shape emotions and horizons of possibility while reflecting on the broader socio-political implications of this phenomenon. Before turning to this, in the following sections, I provide an overview of the material context shaping emotional and temporal perceptions of precarity in Spain, as well as the methodological approach I took to facilitate open and sensitive conversations about the future.

## Context: Youth precarity in Spain

In the Global North, the southern European context offers a valuable vantage point for examining the ways in which systemic labour and housing precarity translates into multi-dimensional, lived experiences of ontological precariousness. In Spain, research on employment and transitions into adulthood highlights the persistent prevalence of high youth unemployment rates, temporary employment, low wages, late departure from parental homes, and dependence on a familialist welfare state (Guillén and León, 2011; Moreno, 2012; Verd et al., 2024). Like in other European nations, the late 1970s marked a pivotal juncture in Spain's Fordist order with labour deregulation disrupting and delaying post-war pathways to adulthood (Rueda, 2014; Verd et al., 2024).

Prior to this, the persistence of an authoritarian dictatorship meant that late Fordist development – despite strong economic growth in the 1960s – did not result in the development of a redistributive welfare state. Only from the early 1980s – when welfare institutions were under attack in the West – did Spain develop a welfare state comparable to other high-income European countries, sharply increasing spending on pensions and unemployment, and with a universal healthcare and education system introduced only in 1985 (Guillén and León, 2011). In this period, considerable progress was made in terms of gender equality, with key laws such as equal pay, consensual divorce, and abortion being passed. Spain rapidly moved from having the lowest women's employment rate in Europe to currently being above the European Union (EU) average (Guillén and León, 2011). However, this progressive shift was accompanied by neoliberal labour reforms aligned with global trends, resulting in sharp labour market dualisation, deindustrialisation, and the persistent dominance of a low-productivity, service-based economy (Rueda, 2014). As a result, today temporary workers, disproportionately comprising young people and women, find themselves burdened with lower wages, limited social protection and easy terminations while their counterparts with permanent contracts enjoy robust protection. Unlike in other contexts, stark and persistent labour precarity emerges in Spain at the very moment in which social-democratic institutions were developed. While the post-war period provided stability for a selected number of male workers under a repressive and authoritarian regime, the 1980s experienced both institutional and social progress in a context of growing labour precarity. In this sense, participants’ nostalgic attachments to a perceived past of security discussed below must be contextualised within Spain's broader historical context, where such security has been neither inclusive nor equitably distributed.

Austerity measures following the 2008 crisis profoundly deepened structural youth precarity with the youth unemployment rate peaking at 56.1% in 2013.^2^ Since 2018 the centre-left government has taken important steps to address the enduring effects of austerity. They have notably increased the minimum wage, introduced a minimum income guarantee scheme, and enacted a labour reform that has reduced temporary employment (Verd et al., 2024). Despite this, precarity, casualisation, and low-wage employment persist among young people. Spain currently has the highest youth unemployment rate (29%) among The Organisation for Economic Co-operation and Development (OECD) nations and the highest share of young employees with an involuntary temporary contract in Europe (Eurostat, 2022). Temporary employment often leads to dead-end jobs, trapping young people in instability and increasing the likelihood of dismissal (Verd et al., 2024). Furthermore, the surge of graduates in the last four decades, with 49% of people aged 25–34 holding university degrees (Eurostat, 2022), has resulted in a highly qualified working force. However, as in other European contexts, the mismatch between qualified workers and the availability of suitable jobs leads to underemployment; a phenomenon that pushes graduates into low-paying jobs below their qualifications, worsening prospects for non-graduates (Furlong et al., 2017).

Since 2013, soaring rent prices combined with minimal social housing (2.5% of housing stock) (OECD, 2020) have fuelled a trend where young adults delay leaving their parental homes, with only 16.3% of people aged 16–29 living independently – significantly lower than the EU average of 31.9% (Eurostat, 2022). In Catalonia, where this research was conducted, a young person aged 16–29 on the median income would need to allocate over 100% of their monthly net salary solely for rent if they were to live alone (CJE, 2023). Similarly, buying a property would mean saving their entire salary for 4.5 years for a mortgage down payment and allocating three-quarters of their monthly income to mortgage payments (CJE, 2023). In Barcelona, where the average rent peaked at €1171 in 2024 (the highest in Spain), the issue of housing unaffordability is even more acute.

Housing wealth increasingly reinforces inter-generational inequality in Spain, which ranks second in the OECD for inheritance value – largely due to household investment in property. Yet it also has one of the widest inheritance gaps: the wealthiest 20% receive an average of €350,000, compared to just €10,000 for the poorest 20% (Maqueda, 2018). As housing prices rise far faster than wages, private rent has become the fastest-growing form of tenure with tenants increasingly trapped in a dynamic of surging rental costs – a phenomenon no longer confined to the young. The rise in rented households is most pronounced among those aged 30–44 and 45–65, while it has declined among younger cohorts due to delayed departure from the parental home (Montoriol-Garriga, 2023). The trend of housing insecurity affecting older cohorts aligns with participants’ perceptions of precarity as increasingly extending across the life course. Housing and employment challenges influence family decisions, as reflected in Spain's second-lowest fertility rate in Europe (1.16%), only after Malta, and in Spanish women having their first child at 31.1 years, the latest in Europe after Italy.^3^ These underlying politico-economic conditions amplify feelings of future uncertainty among young people and contribute to an increasingly widespread sense of social decline.

## Methods

The interviews were conducted using an innovative qualitative methodological approach – Oral Histories and Futures – developed by Sarah Marie Hall (2024). Oral Histories and Futures expands upon the oral history tradition by introducing a forward-looking lens supported by creative and participatory methods. These methods were used to capture the complexity of life stories by recognising the dynamic interplay between past experiences and future imaginings and aspirations. Interviews, lasted 60–120 min, yielding rich material, including detailed accounts and thick descriptions of the participants’ lives and future imaginings.

In the recruitment phase, I collaborated with two partner organisations in Barcelona: ABD, a third-sector organisation dedicated to supporting vulnerable people throughout various life stages; and PAH, a housing rights organisation. These organisations made calls for participation in their meetings and on social media. I took a participatory approach to recruitment by volunteering in their projects and campaigns. Additionally, I disseminated information through posters which were strategically placed in universities, vocational schools and youth centres. Snowballing also became an important element of recruitment as fieldwork evolved.

Thirty-nine participants aged 19–35 participated in our project: 28 self-identified as women, 7 as men, and 4 as non-binary. It is difficult to classify participants in terms of class due to the important variations in terms of backgrounds, family income, and life trajectory. However, none reported incomes above the Barcelona median gross income (€24,053).^4^ Only one participant owned a mortgaged home in a working-class town on the outskirts of Barcelona; the rest lived with parents or grandparents, rented shared accommodation, or lived with partners in rental housing. Twenty-six participants were university students or graduates, most working part-time, and some full-time, while studying – tuition fees in Catalonia average €1200, with grants available for low-income households. Of the participants, 13 were in full-time employment, 19 were in temporary, part-time employment, and 7 were unemployed. In terms of country of origin, 25 participants were born in Spain, 7 in Latin America, 5 in other European countries, and 2 in Morocco. Participants were given the option of anonymity; hence, some names in the paper are real while others are pseudonyms.

During the interviews, participants were invited not only to share their past life stories but also to reflect on how they shape their present experiences and future trajectories. I incorporated participatory creative elements to deepen engagement and reflection. I used two future-scoping techniques: biographical mapping and writing a post-card to one's future self. Biographical mapping was conducted alongside interviews to provide participants with a visual and tactile means to map out both their expected and ideal futures articulating their longings, desires and aspirations. The maps contained different layers that reflected both realistic and desired futures. Participants were encouraged to map the geographical places and homes they envision inhabiting at various junctures in their futures. They were also prompted to delineate the professional activities they see themselves engaging in at different stages of their lives, as well as the significant people they anticipate sharing their lives with and the leisure activities they envisage beyond work. The post-cards aimed to cultivate non-linear future narratives by prompting participants to envision themselves in their later years and correspond with their present selves enabling them to contemplate their life trajectories from a future perspective back to the present. In a context where participants generally hesitated to discuss and envision the future, these activities sensitively facilitated and extended such conversations.

To analyse the data, I employed a thematic analysis approach, coding interviews, maps, and post-cards to identify recurring patterns, tensions, and divergences in participants’ aspirations and imagined futures. The mapping and post-card data were integrated into the analysis by examining the spatial and temporal dimensions of participants’ envisioned trajectories examining not only the constraints and possibilities they articulated through these creative exercises but also their emotional response to the future. These visual and textual materials provided a complementary layer to the interview data, revealing implicit themes and affective dimensions of participants’ future orientations that might not have surfaced in verbal narratives alone.

## The multi-dimensional experience of urban precarity

For the participants in this research, systemic labour and housing precarity were not only experienced as present conditions but also as anticipated futures, producing a pervasive sense of uncertainty that redefines urban life and its meanings. The following narrative stands as poignant illustration of the spatial and multi-dimensional character of precarity. Paula – a 34-year-old Spanish woman currently unemployed after experiencing severe burnout – described the intense demands of her previous job in the third sector. She explained that the lack of boundaries around work and life, combined with constant housing insecurity, not only left her physically and emotionally depleted but also distanced her from her family and loved ones. She referred to the difficulties of envisioning a prosperous future in Barcelona:

Barcelona has always been one year, moving house, another year, moving house, a few months, moving house. I've always lived with the feeling that I'm here temporarily. Deep down, I always felt certain that this will end at some point and I'll have to go back to my village. So that's why on a professional level I've never imagined anything that would be the job of my life, even on a personal level with people or partners. I find it very difficult to say ‘I see myself in the future with this person or in this job or in this house’.

Paula's lived experience illustrates how the intersection of labour and housing precarity shapes experiences of acute ontological insecurity, weaving into participants’ temporal landscapes. This resonates with conceptualisations of precarity as a state of uncertainty characterised by one's inability to predict (Ettlinger, 2007; Rossiter and Neilson, 2005). In the case of Paula, instability and temporality hinders their ability to nurture long-term personal relationships in place. This aligns with research highlighting how labour precarity can lead to reduced social interactions and the erosion of relationships (Woodman, 2013), illuminating the multi-dimensional character of precarity.

The level of uncertainty, work insecurity, and acute exploitation experienced by some participants has shaped their ability to plan, disrupting the most intimate aspects of their lives. Laura's (26 years old) narrative illustrates of how labour precarity sips into intimate life. Having endured the unbearable experience of signing 18 contracts in her first year working as a nurse amidst the COVID-19 crisis,^5^ she found herself grappling with an acute state of burnout, leading to a depression which required some time off work and psychological support. Extreme work precarity has affected her ability to plan:

If I have a job where they make me sign month by month, well, maybe I can’t commit to starting a family because I have another mouth to feed. I do need job stability, so I don't know, I'll just go with whatever comes.

Diana, a 26-year-old, was forced to return to her mother's home in a small town in Girona after 6 years of independent living in Barcelona, unable to keep up with the relentless rent hikes. She is currently completing her degree in physics while working in the hospitality sector. Diana has long grappled with the desire to start a family. However, her recent return to her family home compounded by the ongoing precarity experienced by her partner has prompted her to reconsider this.

My father began working at La Caixa (a Spanish bank) when he was 17 years old and worked there until he was 55, so he never changed jobs. My partner has changed jobs about 8 times. So … if you’re aware of that, and you're aware that life costs a lot of money, that children cost a lot of money, well, little by little that idea of ‘maybe I won't have children’ has been creeping in.

She added that ‘emotionally I would like to have children, but economically it is very complicated’. In the case of Diana and Laura, the absence of labour and housing stability in the present alters and forecloses certain family futures.

As illustrated by the following narrative, the persistent labour and ontological insecurity can erode the power of governmental precarisation. Lucía, a 31-year-old first-generation graduate with an adventurous spirit, spent her 20s living in different countries and doing multiple jobs while completing one degree in anthropology and two master's degrees. In her early 30s, she returned to the Barcelona Metropolitan Area seeking some career progression. Here, she endured an intense experience of burnout while working for a third-sector organisation, where long hours took a toll on both her personal life and mental health. In this period, she also struggled with diverse experiences of housing precarity, leading her to temporarily live in a campsite where she had spent holidays as a child. After this period, she finds herself surprised by her new longing for labour, housing, and family stability.

It scares me even to think that I long for security because it's a bit contrary to how I thought I was. Or maybe if the state provided and had some confidence in public policies that allowed me to live in a more disorganised way, I wouldn't mind … But I know that all the responsibility falls on oneself.

Experiences of intense and prolonged precarity have led Lucía to long for a type of security she once rejected. Like other participants, this stems from disillusionment with pursuing – or being trapped in – forms of flexible life that ultimately lead to ontological insecurity and emotional depletion. This narrative encapsulates how the denial of material security through empty promises of autonomy has undermined the power of neoliberal flexibility, transforming post-war notions of stability into a longed-for yet unattainable desire. In this sense, participants neither fully engage in self-precarisation nor succumb to the self-responsibility often associated with the damaging effects of cruel optimism (Pettit, 2019). In our conversation, Lucía also shared that, at this stage in her life, she wants to find a way to make having children possible. Despite her challenges with housing in the Barcelona Metropolitan Area, she also felt a strong need to settle geographically to meet her caring responsibilities towards her parents and loved ones. However, as Lucía and other participants noted, there was a widespread perception that, when it comes to making their desires for stability and security viable, ‘all the responsibility falls on oneself’. Far from leading to self-blame, participants’ critical responses to the individualisation of responsibility reveal a reframing process that shifts accountability from the individual to the structural failures of the neoliberal state.

As seen in the narratives above, the enduring lack of continuity across multiple facets of life has reshaped participants’ relationship with Barcelona and, more broadly, with urban space. Most of them do not articulate their longings for a better life through the seductive power of neoliberal notions of autonomy, temporariness, and flexibility (Ferreri, 2021). Returning to the discussion above, this appears to weaken the seductive appeal of autonomy and flexibility central to neoliberal urban discourses while driving imaginaries of the future away from the city. The city is no longer experienced as a realm of boundless possibilities where dreams and ambitions – however fragile or cruel – take flight. This both aligns with and diverges from Ferreri's spatialised notion of precarity, linked to the ambivalence of the temporary city, where young people embrace the fleeting social advantages of flexible living while grappling with anxiety over the lack of continuity. While participants expressed feelings of anxiety, they did not appear to be drawn towards the temporariness of the neoliberal city as a space of leisurely enjoyment. However, echoing Ferreri's (2021: 165) notion of impotence as ‘the self-imposition of limits and limitations to action projecting onto territory and spatio-temporality in the future’, the city no longer functions as a horizon of future-oriented hope and potentiality. Instead, the urban is perceived a battleground where endurance and survival are the only certainty. As in the case of Paula above, who felt certain that at some point she would need to return to her village in central Spain – paradoxically, an area profoundly affected by economic decline and depopulation – many participants envisioned similar futures in their biographical maps, imagining a return to rural areas or smaller towns, or seeking a quieter life beyond the city.

Housing unaffordability was identified as key factor amplifying uncertainty and insecurity, undermining participants’ ability to see Barcelona as a viable place to build a life. Júlia, a 19-year-old, history student who works part-time as shop-assistant in a fast fashion shop, illustrates a feeling shared by most participants:

Barcelona isn’t for those of us who live here. Barcelona is a pretty showcase for tourism, where tourists come to live – and with foreign incomes, you can come here and afford to rent a flat on Airbnb and pay €1,600 in rent. But what is someone from here supposed to do? … When I’m earning minimum wage, more than half of my salary would go just into renting a room, you know? It's really tough, and honestly, my expectations for the future are kind of bleak, I won’t lie. It's like, ‘what am I going to do?’ I’ll be living with my grandparents until I’m 30.

The city was neither described as a place where personal and professional development was possible, nor was its seductive appeal sufficient to anchor the participants’ future imaginings within it. As Victor, a 33-year-old associate university lecturer (*professor asociado*) who has been working on temporary contracts below the minimum wage for the last 5 years, described^6^:

The city is full of bad smells, noise, and people rushing everywhere with unfriendly looks. I think it also has to do with the current situation – we’re in a tough spot, people are struggling a lot, and they don’t see things getting better. It's like the 2008 financial crisis, and just when it seemed things were starting to improve, boom – pandemic, economic crisis, inflation … and it just keeps piling up.

Urban precarity – understood as a multi-dimensional phenomenon shaping material conditions, life-course decisions, relationships with space, emotions and temporalities – drive young people's future imagining away from the city (regardless of whether non-urban life is materially feasible), foreclosing the possibility of envisioning hopeful urban futures. As I go on to discuss, the temporal–affective structure emerging from the anticipations of precarity fosters a sense of inevitable social decline and loss, reconfiguring the emotions and temporalities tied to cruel optimism.

## Unending precarity: Loss and social decline

Many of the participants articulated their visions of the future through the lens of permanent insecurity which amplified feelings of social decline. They grappled with the sobering realisation that their lifeworlds may be on a trajectory of incremental deterioration. When I invited the participants to articulate their ideal life trajectories through the biographical maps, they commonly cited yearnings for traditional forms of employment, homeownership, and given these conditions, parenthood as part of that imagined future. However, they were highly sceptical of achieving that type of security and stability. At most, a small group of participants imagined achieving some degree of individual stability through the potential inheritance, not of their parents’ homes, but of their grandparents’ – reflecting the growing role of inter-generational wealth in pathways out of precarity. Yet, beyond their specific personal circumstances, they all shared a pessimistic view of society's overall trajectory.

A prevailing sentiment among them was the anticipation of downward mobility, especially when contrasted with the life trajectories of their families. Laia, a 23-year-old from a rural area in northern Catalonia, who moved to her grandparents’ house in Barcelona to study geology, eloquently captures this sentiment. At her grandparents’ home, Laia shares a small room and sleeps in the same bed with her older sister, who had also previously relocated to Barcelona to study, as they can’t afford to rent a room in a shared house. Below, she refers to a generational shift, comparing the perceived upward trajectory of their parents and grandparents with their own experience. She laments the discrepancy between their youthful energy and what she defines as a rupture with societal progress, which she links to an expanded sense of generational disillusionment:

I sense a sort of, I don’t know if it is indifference … but rather like a kind of feeling disillusioned, because we don't have dreams, we don’t see how we can achieve our dreams (…) I believe that the generation of our parents and grandparents experienced an increase in quality of life since the end of World War II, etc. I think there was an increase in well-being and now, in my opinion, the issue has stabilised, or we have reached a turning point and we see the decline. I have the feeling that we only see the decline, something our parents have not experienced or will not experience in the time when you are supposed to be most eager to take on the world, which is when you are young, when you are starting to shape yourself as a person. So, of course, it's a bit despairing to see that you have so much energy to do so many things and you see that your world is not rising but falling apart.

Similarly, many participants shared a sense of cruel nostalgia for a lost, secure past. These reflections were often based on relational family experiences through which participants compared their experiences of their parents and grandparents with their current material conditions. This echoes Diana's narrative, which suggests that her parents’ generation benefitted from a level of stability that seems unattainable today:

I think it was much easier but I don’t know, I say this because I talk to, well, with older people, people my parents’ age and they don’t remember so much instability or so much trouble.

My father went to live alone when he was 17 years old. And he paid for the flat with what he earned, but he lived alone, how crazy! I mean, I can’t imagine it, I can’t imagine it at all. Now rent for one person is easily 1,200 euros a month for sure, but if that's what you make! It's impossible.

As discussed above, these narratives mark a departure from the emotional–temporal logic associated with cruel optimism, as participants appear to have relinquished hope for attaining stability in the future. This aligns with conceptualisations of precarity as a form of loss (Allison, 2013). However, in this case, loss is experienced not only in relation to stability and security tied to the past, but also as a persistent presence that unfolds across the life course. When I asked Maria – a 21-year-old woman with a working-class background who lives with her parents and is the first in her family to pursue a university degree – how she thinks her generation looks at the future, she also referred to a generational sense of despair rooted in the certainty of downward mobility:

All depressed. I believe that half of my generation is doomed. Or me too, doomed. When you compare yourself, it's ugly to compare yourself, but you compare yourself with what your parents did, you say ‘wow, at 20 they were already married and had a daughter, for example’ … So I don’t know, I think my generation is all very discouraged. Very discouraged by what awaits us, more than anything.

Echoing Hall's (2022) notion of austerity as an enduring – at times seemingly unending – condition, and del Río et al.’s (2025) idea of foreclosed futures, precarity was not seen by most participants as a temporary phase delaying transitions into adulthood. Rather, it was normalised and accepted as a permanent condition. For instance, Cristina (33 years old), who migrated from Colombia to Spain in 2012, described what this feels like:

Well, I can't say because for me it's always been like this, so I don’t know, the opposite I don't know what it is. I mean, I don’t know what it's like to live in a country, let's say, like Canada, I don't know what it feels like to be in a place where there isn’t … Where everyone has their job, earns well, has their own place, where there isn’t this feeling that you’re going to lose everything overnight, like a sword hanging over your head. I don’t know, I’ve normalised it since birth, I mean, I’m like this because I’ve never lived in anything other than this. So, I feel normal, I mean, for me, the abnormal is the opposite, being in a place that isn’t like this.

While participants’ longings for security were temporally placed in imagined secure pasts – or, as in Cristina's case and others discussed in the previous section, in distant geographies beyond Barcelona – most regarded stability as increasingly out of reach, giving rise to a deep sense of despair and disillusionment.

Rather than clinging to dreams that have been structurally denied, the participants’ stories reveal a deep awareness of the material constraints shaping young people's lives. Yet this critical awareness often gives way to resignation and a sense of powerlessness. When I asked Elena – a 19-year-old working-class first-generation university student completing a degree in economics – whether she believed that her career would stabilise at some point, she cynically laughed:

Would I like that? Definitely. Do I actually see it happening? Honestly, not really. The most job stability I can imagine is maybe staying in the same place for two or three years. But I don’t expect things like regular pay rises just because I’ve been at a company for, say, 15 years. That wouldn’t feel normal to me – it would feel more like a fluke.

As this narrative suggests, attachments to Fordist forms of security associated with cruel optimism have not been abandoned as aspirational ideals conforming hegemonic notions of material security. Instead, they are gradually perceived as unattainable, relegated to the past instead of a promising future to pursue. This suggests that the normalisation of precarity as a constant throughout the life course has led to a shift in the temporalities and emotions associated with cruel optimism, transforming them into cruel nostalgia and thus foreclosing generative attachments to the future. As I turn to discuss, instead cruel optimism, disillusionment, hopelessness, and powerlessness were the core emotions expressed by participants, leading to attitudes such as pragmatism, cynicism, and scepticism about the possibility of better future – a shift with far-reaching socio-political implications that demands further exploration.

## Foreclosed futures: Temporal–affective experiences of enduring precarity

As noted above, temporality – and more recently emotions – are key dimensions in geographical conceptualisations of precarity. Precarity has been theorised in relation to the future as both a condition of contingency and unpredictability (Ettlinger, 2007), and as a mode of governance and subjectivation where the anticipation and normalisation of instability erodes potential for imagining alternative futures and other forms of the life (Ferreri, 2021). I develop the idea of foreclosed futures to deepen temporal understandings of precarity through an emotional lens. Echoing European research on emotional geographies of precarity (Marcu, 2019; Raynor, 2021; van Lanen, 2021; Wilkinson and Ortega-Alcázar, 2019), participants’ narratives frequently resorted to a lexicon dominated by terms such as ‘uncertainty’, ‘fear’, ‘instability’, and ‘vertigo’ when referring to the future. The following testimonies convey a sense of foreclosed futures, understood a temporal–affective formation shaped by negative emotions obstructing the imagination of non-precarious alternatives:

Diana: Uncertainty, quite a bit of uncertainty, and not the good kind of uncertainty, but more like, ‘I don't know what we are going to do.’ I try not to focus too much on the future because it's not something I think positively about … You know? I focus a lot on the present and taking things step by step, and we'll see where life takes me.

Alessia: All my friends are more or less like me; they are very uncertain about what is going to happen. You don't know what's going to happen, so it seems to me that my generation has stopped planning.

Lucía: Uncertainty, uncertainty, and at the same time it worries me, and I feel bad that this is what comes to mind when thinking about the future. But the first word that comes to mind is that, and I would love that the first word was ‘projects’, which would maybe be the second, but it's not the first (…) And that feeling of not having security and not being able to plan ahea because you don't know what's going to happen, it's heavy.

All these narratives collectively underscore a palpable fear towards the future rooted in their inability to predict, grounding participants firmly in the present, with their hopes and dreams held captive by uncertainty. This ‘live in the moment’ attitude taking by participants implies a response to the looming spectre of precarious future, driven by the imperative to focus exclusively on immediate survival. As Berlant (in Rand, n.d.) observes in a late discussion about what she recognises as the diminished power of cruel optimism in contemporary society: ‘One of the things that happens in this model of entrepreneurial subjectivity is that survival looks like the best possible horizon of living … And survival is success. It's so terrible that people's best creative energy is sucked up trying not to drown’. This profoundly resonates with the affective structures that foreclose participants visions of alternative futures leading to present-focused attitudes and survival strategies. When I asked Laia how about labour market demands, she echoed this dynamic:

Yes, obviously there’s this feeling that you must do your utmost, you must give the maximum to have any opportunity. I mean, my outlook is very pessimistic in that I see there’s a lot of competitiveness and you must be very well-educated to have any chance. So yes, I must do many things to feel calm or to guarantee myself a place.

Laia’s hard work is directed not towards hopes of achieving ontological security or neoliberal success, but towards securing herself a job. This partly aligns with Raynor's (2021) insight into how working-class women in austerity England direct their hopes not towards upward mobility, but towards maintaining an already precarious position.

As discussed above, Berlant (2011: 2) gestures towards the socio-political implication arising from the emotional responses tied to the broken promise of crucial optimism: ‘What happens when those fantasies start to fray – depression, dissociation, pragmatism, cynicism, optimism, activism, or an incoherent mash?’ In the narratives shared by participants, pragmatism, cynicism, and scepticism were particularly prominent, revealing a conscious refusal to cling to ‘unrealistic dreams’ to shield themselves from disappointment. Here, Laia again, eloquently encapsulates this:

Throughout all these years I have been building myself realistically, that is, I do not allow myself to have dreams about something that I think is very difficult to achieve, simply as a defence mechanism so that later I do not have disappointments. I do allow myself to think ‘oh, imagine this, it would be …’, but I know it will not happen, so … And I also don’t believe it's something I need, so I always maintain a balance between what I would like and what I believe could happen, and this is where I stay. It's always a bit about finding the middle ground, staying grounded.

This narrative illustrates how normative aspirations for security and stability are increasingly perceived as unachievable, prompting a shift towards pragmatic strategies and expectations.

Like Laia, many participants made or were willing to make sacrifices in pursuit of (precarious) jobs aligned with their passions. Others, however, exhibited a more sceptical and cynical outlook, harbouring minimal hope for career progression. The following testimonies illuminate how some of the participants felt trapped in an endless cycle of low-paying jobs which demanded sacrificing great amounts of time and energy merely to meet basic needs. As Irene, 23-year-old from Barcelona and of Ecuadorian descent, has predominantly worked in the hospitality sector, puts it:

There are these precarious jobs out there, and well, in the end, you search and find work, and you keep searching and finding. But it's like there's this loop where you end up trapped in a cycle of work and you reach a limit that you can’t surpass.

Alessia (25 years old), who moved to Barcelona from Italy and has primarily worked in call centres combining periods of unemployment, referred in similar terms to the disposable character of precarious jobs:

It doesn’t make sense to stick to a stupid job for years, which is going to pay you next to nothing. We’ve realised it's pointless, so it's like, you have to be smarter about it. We’ll stay here for a bit until it suits us, then move on to another place where we feel a bit better, and so on. I see us taking everything as fast food, even with our jobs.

When discussing old-age security, through the issue of retirement and pensions, several participants expressed hopelessness, cynicism, and resignation as well as a lack of trust in institutions. Below, Manuel, a working-class university student who migrated to Catalonia from Argentina at the age of two, captures these feelings:

I think there's a lot of hopelessness, especially in the sense that, even when talking about pensions, many people my age are not sure if we will have pensions, and we say it maybe with a somewhat cynical tone, in the sense of ‘we don’t care,’ but we have no idea what the future holds for us. I believe a possible economic collapse is looming, and I don't know, I suppose we have to take it in the best possible way.

While Laia's pragmatic approach and Irene's, Alessia's, and Manuel's cynical and sceptical outlook demonstrate awareness of the societal constraints, they also suggest the acceptance and naturalisation of such constraints as permanent features in their life courses. This creates a paradox where young people assert agency by recognising structural constraints and resisting self-responsibilisation. However, instead of fostering collective empowerment and prefigurative imagination, this form of critical agency leads to a sense of powerlessness and impotence – the latter, as Ferreri puts it (2021: 165), not only ‘understood as a lack of power but rather as the absence of potentiality’. Perhaps, as precarious life becomes increasingly normalised and scepticism and cynicism deepens, the ability to form affective attachments to alternative visions of the good life – more inclusive, internationalist, and egalitarian than those tied to the post-war settlement – may also fade. This, I argue, is what the idea of foreclosed futures encapsulates: not only the projection of current structural barriers to upward mobility into the future, but also a temporal–affective structure that precludes the formation of generative attachments to alternative visions of the good life. The final section draws together the paper's core contributions while reflecting on the broader implications of foreclosed futures and the political mobilisation of nostalgia.

## Conclusion and reflections on the politics of nostalgia

This paper contributed to geographies of precarity by advancing multi-dimensional framework rooted in the temporalities and emotions emerging from enduring insecurity. It revealed how enduring precarity produces temporal–affective formations that reconfigure aspirations, horizons of possibility and spatial belonging. First, by advancing a multi-dimensional conceptualisation of precarity, I explored how lived experiences of insecurity projected into the future influence personal relationships, family decisions and the meaning of urban space. Lived housing and labour insecurity precluded the cultivation of hopeful, affective attachments to the urban, disrupting any hope for continuity and constancy, thus closing off visions of non-precarious futures. Instead of viewing the city as a space of boundless possibility and upward mobility – however hollow or cruel those promises and expectations – many participants envisioned leaving Barcelona for more peaceful, affordable places. In this sense, the seductive power of neoliberal city, which is often described as a key driver of self-precarisation, has failed to anchor young people's future aspirations and dreams in the neoliberal city.

To develop this argument, I built on del Rio et al.'s (2025) concept of foreclosed futures, advancing it as a temporal and emotional dimension of persistent urban precarity. I showed how the anticipation of unending insecurity by young people in Barcelona transforms the emotions of temporalities linked to cruel optimism into what might be called ‘cruel nostalgia’. On the one hand, post-war notions of the good life continue to function as the grammar through which security is imagined and understood. On the other, this grammar is articulated solely in the past tense, no longer as a cruel attachment fuelling hope for better futures, but as a nostalgia for a past irretrievably lost. Finally, I showed how precarious entanglements – where the material, affective, temporal, and spatial become embodied – shape the horizons of possibility, potentiality, and futurity. The anticipation of an insecure future evoked emotions such as fear and anxiety, tethering participants in present-oriented survival strategies. I argued that hopelessness for a better future is rooted in the exercise of agency through a critical awareness of present structural barriers and a refusal to internalise blame. Yet this agency, rather than enabling collective imagining or future-oriented action, breeds a debilitating sense of impotence – understood as lack of potentiality.

I conclude by briefly reflecting on the potential political implications of foreclosed futures, understood not only as the internalisation of precarity as a permanent state but also as the affective and temporal closure of alternative life possibilities. The pragmatic, sceptical, and cynical dispositions emerging from foreclosed futures close off the possibility of generative attachments beyond those tied to cruel optimism. Notions of the good life inherited from the post-war era are seen as illusions betrayed by the precarious present, yet they still haunt the present as a nostalgic yearning for a time when life seemed more secure. In this sense, attachments to post-war notions of upward mobility continue to inform hegemonic ideals of the good life, which are now imbued with a sense of nostalgia.

Although this was by no means the case for the participants, the impotence associated with foreclosed futures is becoming fertile ground for exclusionary politics driven by nostalgia. In a political economy marked by uncertainty and institutional distrust, authoritarian politics are resurging, often mobilising nostalgia as a political tool to fuel a renewed form of exclusionary cruel optimism. Geographically centred slogans like ‘Make America Great Again’ – adopted in Spain by the far-right party Vox and more recently by the Patriots for Europe bloc – evoke a romanticised past to legitimise exclusionary agendas, fostering nativist calls for a ‘better future’ that mask ongoing processes of upward redistribution. They also suggest that the experience of foreclosed futures transcends the geographical scope of this paper. As these narratives gain increasing popularity among younger people (Cokelaere, 2024), socially marginalised groups – migrants, women, trans people, and religious, linguistic and ethnic minorities – are cast as scapegoats, portrayed as those usurping the good life. These political discourses reinforce the exclusionary dimensions of post-war Fordism while suppressing its more universalist elements, linking cruel promises of secure futures to ethno-nationalism, anti-migration politics, and a reassertion of traditional gender roles.

In considering how we might move beyond the void left by the materially denied – yet persistent and increasingly socially damaging – attachments tied to cruel optimism, Berlant (in Rand, n.d.) poses a thought-provoking question: Are there other concepts of the good life that would be more satisfying than the ones you have been trained to pay attention to? I conclude by addressing this question indirectly. In these turbulent times, looking to the past can also uncover fulfilled and unfulfilled experiences of inclusive politics, policy, and prefigurative imagination – both within and beyond the post-war period – inviting us to reflect on how these legacies might inform unrealised possibilities for the future. History offers a rich archive of imaginaries, struggles, and lived experiences that can help us reconceptualise concepts such as security and work – not as ends in themselves but as means to flourishing lives. At a time when the future feels increasingly foreclosed, those who have critically engaged with or lived through such moments bear a responsibility to transmit their legacies. As researchers – and especially as educators – we play a vital role in transcending recurring iterations of cruel optimism and emerging cruel nostalgia, unlocking reservoirs of imagination that nurture hopeful and transformative futures for younger generations.

## Notes

1.
https://www.socialsciences.manchester.ac.uk/morgan-centre/research/austerityalters/

2. Instituto Nacional de Estadística data.

3. Eurostat data.

4. Ajuntament de Barcelona data.

5. In Spain, 47.2% of public healthcare workers are employed on temporary contracts, with the widespread use of micro-contracts being a common practice (Martínez and Carmen, 2023).

6. In Spain, *profesores/as asociados/as* are part-time, temporary lecturers earning low wages – typically between €400 and €700 per month. In Barcelona, they represent 40% of all teaching staff (Aunión, 2020).
